# Editorial: Remodeling in cardiometabolic diseases: Towards biomarker characterization, target therapy identification, and drug delivery strategies

**DOI:** 10.3389/fphys.2023.1161720

**Published:** 2023-03-01

**Authors:** Hania Ammar, Nivin Sharawy, Christian Lehmann

**Affiliations:** ^1^ Department of Physiology, Faculty of Medicine, Cairo University, Cairo, Egypt; ^2^ Department of Pharmacology, Faculty of Medicine, Dalhousie University, Halifax, NS, Canada

**Keywords:** remodeling, cardiometabolic, biomarkers, targeted therapy, drug delivery

## Introduction

Remodeling in Cardiometabolic Diseases: Towards Biomarker Characterization, Target Therapy Identification and Drug Delivery Strategies is part of the Frontiers in Physiology–Research Topic series. Cardiometabolic diseases represent significant chronic health conditions and have reported to worsen the outcomes of the recent pandemic, COVID-19. They are interrelated diseases including but not limited to hypertension and heart failure. The Research Topic showcases a set of excellent research papers aiming to provide a clear message for the readership of the journal. The submissions cover areas from pulmonary hypertension to vascular animal models and cardiotoxicity.

## Pulmonary hypertension

Pulmonary hypertension (PH) is a complex and progressive disorder, characterized by remodeling of pulmonary arteries. In spite of major advances in pharmacotherapy, PH still leads to premature death. There is still a lack of knowledge about the pathophysiology of PH. Two contributions to this Research Topic address the molecular mechanisms of pulmonary hypertension (Wang et al. and Tan et al.) identifying pathway genes from expression data and future possibilities of gene-based therapies in PH. Wang et al. analysed microarray data from patients with chronic thromboembolic pulmonary hypertension (CTEPH). The authors cultured vascular smooth muscle cells (VSMCs) and observed that TNF-α promoted the proliferation and migration of VSMCs *via* upregulation of FOS expression. Tan et al. examined mRNA-Seq data from different pulmonary hypertension patients with CTEPH or idiopathic pulmonary artery hypertension (IPAH), and compared the results with gene expression profiles of healthy controls. The authors found 76 overlapping genes in downregulated DEGs and 44 in upregulated DEGs between IPAH and CTEPH. Although GTF2H2B, FOS, and FAM114A1 are overlapping genes, they were differentially expressed among CTEPH and IPAH patients. Samples derived from CTEP patients showed an upregulation of FOS and FAM114A1 and downregulation of GTF2H2B. However, upregulation of GTF2H2B and downregulation of FOS and FAM114A1 was reported in IPAH group.

## Vascular animal models

Heart failure (HF) is a disease that is characterized by cardiac remodeling.

Transverse aortic constriction (TAC) is a commonly used experimental model to simulate pressure overload-induced, progressive HF. The model induces reproducible cardiac hypertrophy and fibrosis. However, severity of remodelling varies depending on animal strain/species, sex and genetic background. To which extend these variables affect the severity of cardiac remodelling is yet to be fully tested. In this issue, Huang et al. aimed to compare the effects of TAC in ICR and C57BL/6J mice, using echocardiography, organ index, and histological analyses of the hearts. The authors suggest that ICR mice are eligible for studying pressure overload-induced HF. A reduction in ejection fraction (EF), severe lung congestion, higher Fn1 expression, as well as poor survival rate, support their conclusion.

## Cardiotoxicity

Chemotherapy-induced cardiotoxicity has been reported to be correlated with unfavourable prognosis in cancer patients. In the doxorubicin model of cardiotoxicity, Hanna et al. investigated the effects of melatonin and deferoxamine on cardiac performance. They found that melatonin could promote the action of deferoxamine to mitigate the doxorubicin-induced ferritinophagy and oxidative stress. They observed a significant reduction in the expression of ferritinophagy-inducing genes (NCOA4 and IREB2) and malondialdehyde (MDA) levels after treatment with melatonin and deferoxamine. Meanwhile, a remarkable increase in ferritinophagy inhibitors (SLC7A11 and FTH1), glutathione peroxidase 4 (GPx4), and glutathione (GSH) were noted in groups treated with melatonin and deferoxamine, in comparison to untreated animals. The study highlights the potential benefit of combined strategies to reduce the toxicity of doxorubicin.

